# Reporting and connecting cell type names and gating definitions through ontologies

**DOI:** 10.1186/s12859-019-2725-5

**Published:** 2019-04-25

**Authors:** James A. Overton, Randi Vita, Patrick Dunn, Julie G. Burel, Syed Ahmad Chan Bukhari, Kei-Hoi Cheung, Steven H. Kleinstein, Alexander D. Diehl, Bjoern Peters

**Affiliations:** 1 Knocean Inc., Toronto, Ontario Canada; 2 Division for Vaccine Discovery, La Jolla Institute for Allergy and Immunology, La Jolla, CA USA; 3 ImmPort Curation Team, NG Health Solutions, Rockville, MD USA; 4 Department of Pathology, Yale School of Medicine, New Haven, Connecticut USA; 5 Department of Emergency Medicine and Yale Center for Medical Informatics, Yale School of Medicine, New Haven, CT USA; 6 Interdepartmental Program in Computational Biology and Bioinformatics, Yale University, New Haven, CT USA; 7 Department of Biomedical Informatics, Jacobs School of Medicine and Biomedical Sciences, University at Buffalo, Buffalo, NY USA; 8 Department of Medicine, University of California San Diego, La Jolla, CA USA

**Keywords:** Cell type, Standards, Gating definitions, Human immunology project consortium, Immunology database and analysis portal protein ontology, Cell ontology, HIPC, ImmPort

## Abstract

**Background:**

Human immunology studies often rely on the isolation and quantification of cell populations from an input sample based on flow cytometry and related techniques. Such techniques classify cells into populations based on the detection of a pattern of markers. The description of the cell populations targeted in such experiments typically have two complementary components: the description of the cell type targeted (e.g. ‘T cells’), and the description of the marker pattern utilized (e.g. CD14−, CD3+).

**Results:**

We here describe our attempts to use ontologies to cross-compare cell types and marker patterns (also referred to as gating definitions). We used a large set of such gating definitions and corresponding cell types submitted by different investigators into ImmPort, a central database for immunology studies, to examine the ability to parse gating definitions using terms from the Protein Ontology (PRO) and cell type descriptions, using the Cell Ontology (CL). We then used logical axioms from CL to detect discrepancies between the two.

**Conclusions:**

We suggest adoption of our proposed format for describing gating and cell type definitions to make comparisons easier. We also suggest a number of new terms to describe gating definitions in flow cytometry that are not based on molecular markers captured in PRO, but on forward- and side-scatter of light during data acquisition, which is more appropriate to capture in the Ontology for Biomedical Investigations (OBI). Finally, our approach results in suggestions on what logical axioms and new cell types could be considered for addition to the Cell Ontology.

## Background

The Human Immunology Project Consortium (HIPC) [1] is a multicenter collaboration aimed at performing large studies to profile human immune response to natural infection and vaccination [2]. Each center submits their data to the Immunology Database and Analysis Portal (ImmPort) [3], the primary resource funded by NIH NIAID DAIT to capture such human immunology studies [4]. As part of the HIPC data standards committee, we are working towards harmonizing how data from different centers are represented that describe cell sorting strategies and frequencies of cell populations. We examined studies deposited into ImmPort in the past, focusing on the two fields that describe 1) the cell population targeted, which contains the human language name of the cell type the researcher was studying (e.g. “activated B cell”) and 2) the gating strategy applied, which specifies what markers were being used to identify cells of that type (e.g. “intact/lymphocyte/CD3−/CD14−/CD19+/CD38+”). These two fields in ImmPort are not meant to provide a fully reproducible description of how experiments were conducted, which is captured in other standards such as MIFlowCyt [5] or Gating-ML [6], but rather are intended to reflect how immunologists typically describe targeted cell populations in journal articles and allow one to query for experiments in which cell populations of interest were studied.

An initial examination of the data submitted to ImmPort revealed that, in the absence of precise standards on how these fields should be populated, users have chosen different approaches to populate them. While these are mostly understandable to human experts, they are hard or impossible to parse computationally. Without such parsing, it is not possible to formulate precise queries to identify and cross-compare cell populations from studies across HIPC or ImmPort. We describe here our attempts to standardize the “cell population” field, by mapping it to terms in the Cell Ontology [7] while allowing for additional subset information, and mapping the “gating strategy” field to the Protein Ontology [8] where specific protein markers are utilized. Thus, we hope to standardize how these data are captured utilizing ontology terms. As a result of this work, we propose a new convention on how these two fields should be populated that allows one to interpret them computationally, query across them, and to utilize logical axioms in the Cell Ontology to check for consistency between them.

## Methods and results

### Parsing the gating definitions field

#### Separators

The gating definitions we extracted from ImmPort typically consisted of a list of markers alongside a classification specifying if presence of the marker was detected or not (and in some cases, how strongly), with separator characters such as whitespaces, commas, semicolons, slashes, colons, or any combination thereof being used to distinguish between individual gates. Gating definitions submitted by the same center typically followed the same conventions for separation. We thus implemented center-specific code to split the gating definition into a series of individual gates. For example, “singlet/CD14−/CD3+” is a gating definition from a center using slashes as separators which consists of three gates: “singlet”, “CD14−”, and “CD3+”. While it was mostly possible to re-construct center-specific conventions for separating gates given the large volume of data we had to work on, a general convention on how to separate gates should be agreed upon to avoid ambiguity. Based on our inspection of potential clashes with characters contained in protein names and other markers, we suggest using commas as separators, which were not part of any marker names we encountered, and that rarely occur in generalized names for markers in the Protein Ontology (PRO). To allow for the (unusual) case of specifying marker names that contain a comma, the marker name should be contained within double quotes.

#### Synonyms

Most individual gates name a marker that is detected, typically a protein. The Protein Ontology (PRO) contains a comprehensive list of protein names and accurate synonyms, such as the CD nomenclature which identifies “CD279” as a synonym of “PDCD1”. While a large number of gates could be parsed using the existing PRO nomenclature, we also identified a number of cases where markers were labeled differently than the official labels or exact synonyms in PRO, for example, different capitalization and the presence or absence of hyphens for “KI-67” (“Ki-67”, “Ki67”, “ki67”) or “PD1” instead of “PDCD1”. Table 1 lists examples of proposed new synonyms to be submitted to PRO for consideration as exact synonyms used by the immunology community. Frequently, the proposed new marker labels are lacking hyphens, which are easily confused with the marker being ‘negative’, and thus tend to be avoided when describing markers for flow cytometry. We suggest submitting synonyms to PRO rather than maintaining a separate list for our purposes to recognize PRO as the central arbiter and translator of protein names and synonyms that are themselves based on UniProt standardized names, which will be useful also in other contexts outside of flow cytometry.

**Table 1:** Examples of alternative labels for protein markers commonly used in flow cytometry that will be submitted to PRO

Preferred label in PRO	Alternative label for Flow Cytometry
BDCA-2	BDCA2
KLRB1	CD161
MKI67	KI67
PDCD1	PD1
TNF-a	TNFa

#### Protein subunits vs complexes

While the synonyms in Table 1 were considered not problematic, we also found a number of labels that could not be mapped precisely to individual proteins in PRO. A common case were protein complexes: Investigators often refer to “CD8”, which is a complex of a CD8 alpha and beta-chain, or a complex of two CD8 alpha chains. Unless otherwise specified, gating for CD8+ cells is commonly done by staining for the CD8 alpha chain (CD8A). Similarly, “CD3” usually refers to the invariant subunit of the T cell receptor complex, CD3 epsilon (CD3e), whereas the TCR complex consists of multiple chains. Table 2 lists these cases which we consider to be more problematic. Ideally, all of these protein complexes would be included in PRO, and described in terms of their component chains where possible, but this is not currently universally available. This work should be coordinated with the Cell Ontology, to ensure it matches the logical cell type definitions.

**Table 2:** Markers that could not be precisely mapped to individual PRO protein chains

Label	Description	Suggestion
CD8	A protein complex that serves as a T cell co-receptor consisting of either an alpha-beta heterodimer, or an alpha-alpha homodimer	Use PRO term for CD8 protein complex: PR:000025402. Work with PRO to include alpha/alpha and alpha/beta complexes, and update logical definitions
CD3	The invariant subunit of the T cell receptor complex, consisting of multiple chains including CD3E	Use PRO term for CD3E chain: PR:000001020. Work with PRO to define CD3 complex.
HLA-DR	An MHC protein complex consisting of alpha and beta chains encoded in the DR locus.	Use PRO term for DR beta chain (PR:000036952). Work with PRO to define the different complexes
pSTAT1 pSTAT3 pSTAT5	STAT1/3/5 proteins that have been phosphorylated.	Utilize terms in PRO for phosphorylated forms of proteins (e.g. PR:000003075 for STAT1), and work with PRO to ask if these can be included as synonyms.
LIN	A mixture of lineage markers, typically used to exclude major lineages of cells in blood T cells, B cells, Monocytes, and NK cells, for example: CD3, CD14, CD16, CD19, CD20, CD56.	Disallow this, and ask users to spell out what specific cocktail was used.

#### Other markers

Other examples of proteins that could not easily be mapped are phosphorylated proteins, of which we only encountered three (pSTAT1, 3 and 5), but this and other post-translational modifications of proteins should be handled in a generalizable fashion. Finally, the use of cocktails of markers to include or exclude lineages of cells (e.g. ‘Lin-’) is hard to represent as different cocktails exist, so it would be preferable to spell out what exactly the markers are that are being utilized in a given experiment. Table 2 lists these cases and our current approach to handling them, as well as longer term plans on how to handle them in the future, which will require coordination across several ontology development efforts.

#### Marker intensities

The vast majority of gating definitions consider only positive vs. negative marker patterns, but how they are expressed was variable. Table 3 lists the different marker states we encountered, how they were labeled, and what the preferred label should be going forward. For example, marker negative gating strategies were indicated by adding “-”, “negative”, or “neg” following the marker. Marker positive gating definitions utilized “+”, “positive”, or “pos”. In addition, some gating definitions required qualified variants of ‘positive’, which could be grouped into low vs. intermediate vs. high. In order to enable separating the marker itself from its intensity in a gating definition, we chose preferred labels for marker intensities that avoid alphanumeric characters, as well as avoiding characters commonly used as separators.

**Table 3:** Marker intensities and their preferred labels

Marker state	Preferred label	Alternative labels
Negative	–	neg
Positive	+	Pos
Low	+−	dim, lo
Intermediate	+~	int, medium, med
High	++	bright, hi

#### Size (scatter) based gates

Several gating definitions we encountered for flow cytometry data do not refer to a specific marker, but rather utilize forward- and side-scatter of detector light to provide size estimates of the object passing by the detector. Such gates are commonly utilized to exclude debris and complexes of multiple cells, as well as identify cell types based on size. Additional gating definitions relied on the use of dyes, such as Annexin stains to detect damaged or dying cells that have a ruptured membrane, or CFSE to mark cells that have divided. Table 4 lists the types of values we encountered and the suggested preferred label. Such definitions of cell types based on their characteristics in a flow cytometer are best captured in the Ontology of Biomedical Investigations (OBI) [9], and we are in the process of submitting them there.

**Table 4:** Non-protein based gates

Preferred label	Based on	Alternative spellings encountered
lymphocyte	size (FSC vs SSC)	ly, lymp, lymph, lymphocyte, Lymph, Lymphs, Lymp, Lymphocytes
monocyte	size (FSC vs SSC)	mo, mono, monos, MNC, Monocytes, Mono
granulocyte	size (FSC vs SSC)	Gran
intact	size (FSC vs SSC)	Intact_cells, Intact_cells_population
singlet	relative dimensions (SSC or FSC A vs H, H vs W, A vs W)	sing, singlets, Singlet, Singlets, doublet_excluded, sing-F, intact_singlet
viable	dye	live, Annexin-, live/dead stain-?
proliferated	dye	CFSE-, TracerViolet-

#### Parsing the population name field

When parsing the “Population name” field, many challenges were encountered, with nearly a complete failure to automate this process (< 5% recognition). The guideline to use CL terms for submission of cell populations to ImmPort was found to be insufficient, as most cell populations gated for are further subsets or activation states of CL types. Thus, we revised our approach and developed a future tentative guideline whereby the data provider specifies the parent cell type in CL and adds additional markers or cell subtypes after an ‘&’ symbol. For example, “BDCA3_pmDC” was parsed to ‘plasmacytoid dendritic cell’ & “BDCA3+” and “Temra CD4 T cells” was parsed to ‘effector memory CD4-positive, alpha-beta T cell’ & “Temra”. As a result, the current data set was manually mapped to CL terms.

#### Detecting clashes between population names and gating definitions

The gating and cell population definitions we are proposing express semantics that go beyond simple string labels. Each marker, marker intensity, and cell type is an ontology term, embedded in a network of links to other ontology terms, and to data using those terms. The Cell Ontology uses the Web Ontology Language (OWL) to provide logical axioms for characteristic markers and their expression levels for a given cell type. For example, ‘effector memory CD4-positive, alpha-beta T cell’ is defined as equivalent to “CD4-positive, alpha-beta memory T cell and lacks_plasma_membrane_part some receptor-type tyrosine-protein phosphatase C isoform CD45RA and lacks_plasma_membrane_part some C-C chemokine receptor type 7 and lacks_plasma_membrane_part some interleukin-2 receptor subunit alpha”, linking to the Protein Ontology and the Relation Ontology [10]. These restrictions on the markers known to be present or absent on a specific cell type can be compared to the actual gating strategy used, in order to detect discrepancies that could identify potential errors in data entry, or disagreements how cell types should be defined in terms of markers in the Cell Ontology.

**Fig. 1:**
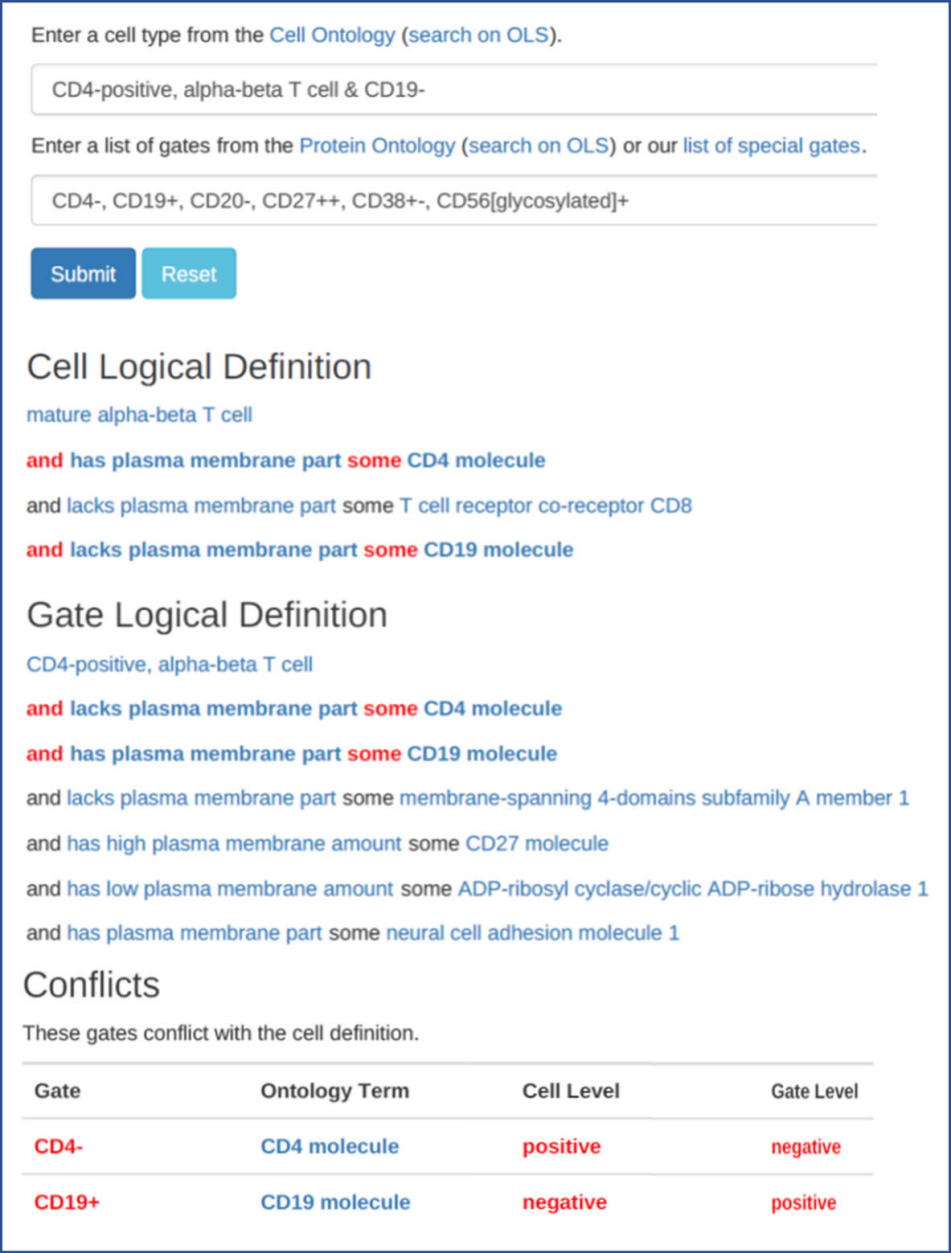
Automated cell field validator. The validator takes a cell type and gating definition as an input, extracts the logical axioms associated with them, and checks for conflicts which are highlighted in red

In order to detect such discrepancies, we implemented a prototype validator, shown in Fig. 1, which can be accessed through https://github.com/jamesaoverton/cell-name-and-marker-validator. The proposed workflow is that users would input “Population name” and “Gating definitions” fields and the validator will check both for validity and cross-check for consistency. This validator can be used to ensure that only valid data is entered into future ImmPort submissions and can also identify discrepancies between the logical definition in the Cell Ontology and what the user is entering. For example, a cell population name and the gating definition conflict, as would happen if the population name was “CD4-positive, alpha-beta T cell” and the gating definition was entered as “CD4− CD19+ CD20− CD27++ CD38 + −” with the conflict being that CD4-positive, alpha-beta T cell are defined as being CD4 molecule positive. In this case, the error is due to the submitted data, but inconsistencies in CL could also be identified, such as the need to define TEMRA cells (a shorthand of TEM cells re-expressing CD45RA) as a distinct sibling of TEM cells (which are logically defined as not expressing CD45RA). We propose to use this approach for a systematic real-life evaluation of cell type definitions and the need for new classes in CL.

#### Application of our scheme to records in ImmPort

We obtained 4388 distinct rows populated from 28 different centers (HIPC projects and others submitted to ImmPort). We began by parsing the “Population name” field with the goal of mapping the entered text to Cell Ontology names. Only a small percentage of these were an exact match to existing Cell Ontology labels, as discussed in the results.

Similarly, the “Gating definition” field was parsed by first “tokenizing” each gating definition by splitting it into distinct gates. Since there was wide variation in the separators and formatting used by different centers, we constructed a set of center-specific rules for tokenizing the gates, primarily as regular expression patterns. The second step was to “normalize” the gate names. We used two strategies: case-insensitive match for the ‘PRO short label’ synonym from the Protein Ontology, and case-insensitive match against a manually compiled list of synonyms described above and shown in Tables 1 and 4. Third, we normalized the marker intensities, as described in Table 3. The overall results are shown in Table 5. We validated the tokenizing and normalizing steps with manual review of the list of gate names by immunologists. The relatively large number of “not matched” gate names indicates both the use of many names and synonyms that were not found in ontologies, and irregularities in gating definition strings. We then wrote a second Python program to demonstrate our vision of the standard terminology going forward as an interactive web page. This tool reads labels and logical definitions from the Cell Ontology, PRO short labels from the Protein Ontology, and our manual list of gate names. The user is asked to enter a cell population name from the Cell Ontology and a set of gates. The tool then displays the logical definition for the cell type and the gating strategy, and highlights any conflicts between the two. Figure 1 shows a screenshot of the tool.

**Table 5:** Protein Ontology short labels accounted for the majority of gate name matches in our dataset with the majority being not matched. Some gate names occurred nearly 2000 times, while others only occurred once

	Gate Name Occurrences	Gate Name Occurrence %	Distinct Gate Names	Distinct Gate Names %
PRO short label	6414	30%	43	14%
exact synonym	2791	13%	44	15%
manual	4283	20%	36	12%
not matched	7722	36%	178	59%
Total	21,210	100%	301	100%

## Conclusion

Here we demonstrated that a vast number of gating definitions, but not cell population names, could be mapped to terms in ontologies, allowing us to semantically express them and detect potential clashes. Based on this, we have identified a number of marker synonyms, protein complexes, and non-marker based gates, and logical cell definitions that we are submitting to the appropriate ontologies (PRO, Gene Ontology (GO) [11], CL and OBI) to further improve such mappings in the future. We have also developed a tentative standard on how to submit gating definitions and cell population names in the future that could be checked for validity and consistency in a fully automated fashion. This standard consists of describing gating definitions by utilizing comma separated PRO terms combined with symbols for intensity (+, −, etc). Size (scatter) based gates are to be captured using a list of preferred terms. Population name is to be described using parent cell type in CL and additional markers or cell subtypes after an ‘&’ symbol. This standard has been tentatively approved by the HIPC steering committee, pending feedback from individual centers. There are a number of limitations to this study that we are aware of: The relationships between “Population name” and “Gating definition” are not absolute, for example if the input sample type is PBMC versus splenocytes (“Population name”) with the same sorting definition (“Gating definition”) the outcome will be different cell types. This means that the validation cannot be expected to catch every potential error and future refinement may be possible after larger datasets are generated using these criteria. We also recognize that our approach is human-centric due to the focus of the HIPC projects, requiring further development if other species were to be included. We were also limited by the fact that that our dataset only had three phosphorylated STAT proteins, thus additional work needs to be done in order to accurately identify post-translational modifications, which will be done in coordination with the PRO representation of such modifications. Lastly, and significantly, implementation of this validation into the ImmPort submission templates is required if it is to be practical and easy for the HIPC community to utilize.

## Abbreviations

CD3e: CD3 epsilon
CD8A: CD8 alpha chain
CL: Cell Ontology
GO: Gene Ontology
HIPC: Human Immunology Project Consortium
OBI: Ontology for Biomedical Investigations
PRO: Protein Ontology
